# Geographical variations in self-rated health and functional limitations among older Chinese in eight WHO-SAGE provinces

**DOI:** 10.1186/s12877-018-1005-y

**Published:** 2019-01-11

**Authors:** Vasoontara Yiengprugsawan, Catherine D’Este, Julie Byles, Hal Kendig

**Affiliations:** 1 Centre for Research on Ageing, Health and Wellbeing (CRAHW), Research School of Population Health, College of Health and Medicine, The Australian National University, 54 Mills Road, Acton, Canberra 2601 Australia; 2 Australian Research Council Centre of Excellence in Population Ageing Research (CEPAR), Canberra, Australia; 3 National Centre for Epidemiology and Population Health (NCEPH), Research School of Population Health, The Australian National University, Canberra, Australia; 4 School of Medicine and Public Health, University of Newcastle, Callaghan, Newcastle, New South Wales Australia; 5 Research Centre for Generational Health and Ageing, School of Medicine and Public Health, University of Newcastle, Callaghan, Newcastle, New South Wales Australia

**Keywords:** Ageing population, China, Functional limitations, Health disparities, Self-rated health, Urban-rural inequalities

## Abstract

**Background:**

The proportion of population ageing in China will grow significantly in the next few decades but the pace of population ageing and social change vary considerably across regions. Notably, Eastern coastal areas are economically more advanced compared to the Western region. These economic disparities could result in differing adverse health outcomes.

**Methods:**

We investigate geographical variations in self-rated overall health and functional limitations in a national representative sample of Chinese aged 50 years and older (*n* = 13,175) using the WHO Study on global AGEing and adult health (WHO SAGE). We used multivariable logistic regression to investigate urban-rural inequalities across regions, adjusting for sociodemographic and health covariates. Two main outcomes were self-rated overall health and functional limitations based on the WHO Disability Assessment Schedule 2.0 for a range of daily activities.

**Results:**

The largest urban-rural differences in adverse health outcomes were in Shandong (AORs for urban versus rural of 6.32 [95% Confidence Interval 4.53–8.82] for poor or very poor self-rated overall health and 5.14 [CI 3.55–7.44] for functional limitations), followed by Jilin (AORs 2.71 [CI 2.04–3.61] and 4.72 [CI 3.43–6.49]), and Hubei (AORs 2.36 [CI 1.82–3.07] and 4.11 [CI 2.80–6.04]), respectively. Covariates significantly associated with both adverse health outcomes were older age, poor income, no health insurance, and increasing number of chronic diseases.

**Conclusion:**

Our study reveals substantial disparities between urban and rural areas observed in both the well-developed areas (eg Shandong) and also the lower end of the economic spectrum (eg Hubei and Jilin). Targeted economic development policy and systematic health prevention and healthcare policies could be beneficial in improving health in later life whilst minimising geographical inequalities.

## Background

Population ageing in China is projected to increase significantly over the next few decades. According to the United Nations (UN) Population Division 2015 report, currently 15% of the Chinese population is aged 60 years and over and this is expected to increase to 25% by 2030 [1]. By 2050 this number is projected to reach 36% of 1.35 billion people, with life expectancy of 82.5 years [1]. The implications of population change and longevity include the rise in non-communicable diseases and subsequent increases of health care needs. These trends present major challenges to the social and health systems to enable older persons to maintain their independence and overall quality of life [2, 3].

Along with sizeable increases in Gross Domestic Product in China in recent decades, income inequalities in large population areas and unequal economic development across the geographical divides are inevitable. Since the modern Chinese economic market reforms in 1978, development in Eastern coastal regions has flourished along with the Western Development Strategy (introduced in 1998), the Northeast Areas Revitalization Plan (2003), and the Rise of the Central China Plan (2009) [4–6]. The Chinese government also began the implementation of medical and health system reform in 2009 targeting universal health coverage, national essential medicine, strengthening primary health care, and reform of public hospitals [7]. The current national 13th Five-Year Plan (2016–2020) further highlights the need to further strengthening medical and healthcare services for the elderly [8].

According to the National Bureau of Statistics of the China Statistical Yearbook 2016, per capita disposable income was highest in the Eastern region (30,655 yuan ~$US4,610) followed by the Northeastern region (22,352 yuan ~$US3,360), Central region (20,006 ~$US3,010), and Western region (18,407 yuan ~$US2,770) [9]. Regional inequalities in wealth and economic development are accompanied by inequality in the distribution of health resources and access to health care services [10–12]. Disparities in life expectancy and health related quality of life have been observed across regions, with longer life expectancy and better health-related quality of life found for people in the more developed Eastern coastal provinces than other regions [13, 14] and in urban relative to rural areas [15–17].

Although there has been increasing evidence on health variation among older adults in China, little is known about the patterns of urban-rural disparities across main provinces. Since regions in China include both major cities and vast rural areas within the same province, differences could be due to rural disadvantage or disparity across provinces. In this paper, we investigate variations in health outcomes among provinces in China using data from a national representative survey of older adults and hypothesise that such inequalities exist between urban-rural areas within each province but the magnitude differs by geographical areas.

## Methods

This study used data from the World Health Organization Study on global AGEing and adult health (SAGE) based on national representative samples of adults aged 50 years and older from a range of low and middle-income countries (China, Ghana, India, Mexico, Russian Federation and South Africa) [18]. China individual data from the WHO SAGE Wave 1 were used for analyses (*n* = 13,175). WHO-SAGE adopted a multistage stratified cluster sample design had similar number of sites per province (4 urban and 4 rural sites each) according to geographic and socioeconomic levels. Four provinces were randomly selected from eastern, two from central and two from western areas (Shanghai, Zhejiang, Guangdong and Shandong; Hubei and Jilin; and, Yunnan and Shaanxi, respectively) [19]. The Appendix provides selected socio-demographic characteristics by provinces based on China Statistical Yearbook 2016 [9].

### Measures and covariates

Two outcomes were of interest: self-rated overall health and functional limitations assessed using the WHO Disability Assessment Schedule 2.0 (WHODAS 2.0) composite measure. Self-rated overall health was dichotomised as poor (‘very poor’ or ‘poor’) or not poor (‘moderate’, ‘good’ or ‘very good’). Poor self-rated overall health has been shown to correlate with mortality [20] and is sensitive to cross-national differences among older adults [21]. The second measure, WHODAS 2.0, covers six domains of functioning, including cognition, mobility, self-care, getting along, life activities, and community participation [22]. Scores within each domain were summed to obtain an overall score ranging from 0 (no disability) to 100 (severe disability) and scores were dichotomised with values ≥25 defined as having some functional limitations [23].

Potential covariates included those known or hypothesised to be associated with study outcomes: socio-demographic attributes (sex, age, years of education, and permanent income quintile). Permanent income was derived from a range of household assets and environmental factors (water, sanitation, cooking facilities) [18]. The permanent income variable is therefore a reflection of income and asset accumulation over time and is a more stable measure than current income.

Health risk factors include current smoking (daily) and alcohol drinking (at least once a week), overweight or obesity (based on body mass index categories using Asian cut-offs) [24], and number of chronic diseases including cardio-metabolic conditions (eg hypertension, diabetes, angina, stroke), arthritis, and depression. Health insurance status was classified according to whether respondents had insurance (mandatory, voluntary, or both) or did not have insurance.

### Statistical analyses

**Fig. 1:**
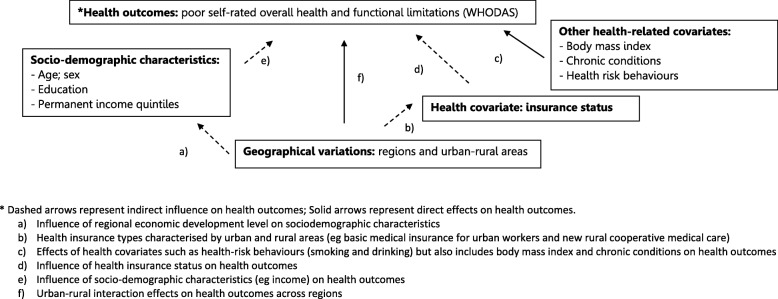
Directed Acyclic Graph specifying conceptual framework for analyses

The analyses were guided by the Directed Acyclic Graphs (DAGs) to describe the conceptual framework for the relationship between outcomes and potential covariates [25]. Figure 1 presents the analytical framework to investigate the relationship between geographical variations and health outcomes, taking into account potential covariates. Multivariable logistic regressions were used to analyse relationships between health outcomes (poor self-rated overall health and functional limitations), adjusting for potential covariates.

Province by location (urban versus rural) interaction terms were included in the models to investigate whether urban-rural differences in outcomes varied across provinces. We have generated estimates for urban/rural comparisons in each province using the main effects and interaction terms from the model. Population weights with post-stratification were applied to analyses using the survey command in Stata [26]. Adjusted Odds Ratios (AOR) and 95% Confidence Intervals [95% CI] are presented.

## Results

In the SAGE sample, Shanghai had the highest percentage of adults aged 70 years and older, and Jilin had the highest percentage aged 50–59 years (Table 1). Females made up to approximately half of the sample. Over 65% of participants had more than 6 years of education across all the provinces with the highest proportions in urban areas, and the smallest difference was seen for Guangdong and Shaanxi. Across all the provinces, Shandong had the largest proportion of obesity (66% in urban areas vs 50% in rural areas). There were large disparities in smoking and alcohol drinking between urban and rural areas across provinces except for Shanghai.

**Table 1:** Distribution of selected sociodemographic and health indicators, WHO SAGE China Wave 1

	Percent distribution (%) by provinces and urban-rural areas^a^															
	Shanghai		Zhejiang		Guangdong		Shandong		Jilin		Hubei		Shaanxi		Yunnan	
	(*n* = 1791)		(*n* = 1463)		(*n* = 1569)		(*n* = 1929)		(*n* = 1702)		(*n* = 1451)		(*n* = 1713)		(*n* = 1557)	
	Urban	Rural	Urban	Rural	Urban	Rural	Urban	Rural	Urban	Rural	Urban	Rural	Urban	Rural	Urban	Rural
Sociodemographic indicator																
Age group																
50–59	54	41	46	52	50	48	53	49	55	54	57	49	52	54	48	55
60–69	23	27	28	26	29	25	23	29	28	26	22	30	27	29	32	25
70+	23	32	25	21	21	27	24	22	17	20	21	20	21	16	20	20
Sex																
Female	51	52	51	46	51	45	51	51	48	52	52	50	49	50	53	47
Years of education																
≥ 6 years	93	66	82	66	73	72	93	66	92	67	86	67	77	73	79	68
Health-related indicator																
Body mass index																
Underweight (< 18.5)	3.3	2.4	4.5	11	3.9	12	0.7	1.4	0.3	2.8	3.8	3.3	3.6	6.8	3.6	6.0
Normal (18.5 to 23.5)	37	32	43	46	29	56	15	26	27	33	35	44	35	52	33	52
Overweight (23.5 to 25.0)	24	22	25	20	23	14	18	22	33	24	22	22	18	17	22	19
Obese (> 25)	36	44	27	23	43	18	66	50	40	40	39	30	44	24	41	22
Number of chronic conditions^b^																
0	41	45	42	45	49	66	43	49	39	55	43	58	46	58	42	55
1	39	32	30	32	32	32	29	32	31	25	34	26	29	27	30	27
2+	28	22	28	22	19	12	27	19	30	20	23	16	25	15	28	18
Health-risk behaviours																
Smoking – current	22	22	17	32	24	33	15	31	22	27	27	33	28	32	24	35
Alcohol drinking – current	14	14	19	31	7.9	29	7.8	20	11	14	15	25	7.0	8.4	11	20
Health outcomes																
Poor self-rated health	9.6	18	11	15	16	18	6	31	13	26	25	40	21	22	21	21
Functional limitations	4.7	7.8	10	6.0	4.7	7.8	5.8	20	5.6	21	7.0	18	14	10	7	18

^a^ weighted % ^b^ Chronic conditions include cardio-metabolic conditions (eg hypertension, diabetes, angina, stroke), arthritis, depression

Hubei, Jilin, and Shandong had the worse rates of poor self-rated health and functional limitations for both males and females. Poor self-rated overall heath was most commonly reported in rural areas. The crude differences in poor self-rated overall health and functional limitations between urban and rural areas were remarkably large in Shandong and Hubei areas.

The multivariable results reported in Table 2 revealed that the odds of poorer health outcomes were higher for rural compared to urban residents for most provinces. The largest urban-rural differences in adverse health outcomes were in Shandong (AORs 6.32 for poor self-rated health [95% Confidence Interval 4.53–8.82] and 5.14 [CI 3.55–7.44] for functional limitations), followed by Jilin (AORs 2.71 [CI 2.04–3.61] and 4.72 [CI 3.43–6.49]), and Hubei (AORs 2.36 [CI 1.82–3.07] and 4.11 [CI 2.80–6.04]). There were moderate statistically significantly higher odds of poor self-rated health for rural compared to urban areas for Shanghai, Zhejiang, and Shaanxi (AORs between 1.5 and 2).

**Table 2:** Explaining geographic variations in adverse health outcomes using multivariable logistic regression analyses, WHO SAGE China Wave 1

Explanatory variables	Adjusted Odds Ratios [95% Confidence Interval]	
	Poor self-rated health	Functional limitations
Rural-urban interaction effects		
Rural Shanghai (*ref:* urban Shanghai)	**1.82** [1.31–2.52]	1.31 [0.85–2.01]
Rural Zhejiang (*ref:* urban Zhejiang)	**1.71** [1.18–2.46]	0.69 [0.45–1.09]
Rural Guangdong (*ref:* urban Guangdong)	0.78 [0.56–1.09]	**3.69** [2.43–5.59]
Rural Shandong (*ref:* urban Shandong)	**6.32** [4.53–8.82]	**5.14** [3.55–7.44]
Rural Jilin (*ref:* urban Jilin)	**2.71** [2.04–3.61]	**4.72** [3.43–6.49]
Rural Hubei (*ref:* urban Hubei)	**2.36** [1.82–3.07]	**4.11** [2.80–6.04]
Rural Shaanxi (*ref:* urban Shaanxi)	**1.55** [1.17–2.06]	1.33 [0.93–1.90]
Rural Yunnan (*ref:* urban Yunnan)	0.97 [0.73–1.29]	**1.88** [1.29–2.74]
Sociodemographic attributes		
Age groups in year		
50–59	*Reference*	*Reference*
60–69	1.07 [0.94–1.23]	**1.61** [1.33–1.96]
70+	**1.41** [1.22–1.63]	**5.25** [4.34–6.35]
Sex		
Male	*Reference*	*Reference*
Female	**1.26** [1.10–1.45]	1.14 [0.97–1.34]
Years of education		
< 6 years	*Reference*	*Reference*
≥ 6 years	1.11 [0.98–1.26]	0.80 [0.68–0.95]
Permanent income quintiles		
Quintile 1 (lowest)	**2.76** [2.18–3.49]	**2.88** [2.09–3.97]
Quintile 2	**1.98** [1.58–2.49]	**2.06** [1.49–2.85]
Quintile 3	**1.71** [1.37–2.14]	**1.83** [1.34–2.49]
Quintile 4	**1.49** [1.20–1.86]	**1.62** [1.20–2.18]
Quintile 5 (highest)	*Reference*	*Reference*
Health covariates		
Health insurance		
Mandatory and/or voluntary	*Reference*	*Reference*
No insurance	**1.22** [1.01–1.49]	**1.36** [1.06–1.76]
Body mass index		
Underweight (< 18.5)	**1.41** [1.12–1.82]	1.16 [0.84–1.61]
Normal (18.5 to 23.5)	*Reference*	*Reference*
Overweight (23.5 to 25.0)	0.92 [0.79–1.07]	0.97 [0.80–1.19]
Obese (> 25)	**0.68** [0.59–0.79]	**1.21** [1.02–1.43]
Number of chronic diseases		
0	*Reference*	*Reference*
1	**2.47** [2.16–2.83]	**1.74** [1.46–2.08]
2+	**4.80** [4.13–5.58]	**3.34** [2.81–3.96]
Smoking		
No	*Reference*	*Reference*
Yes	1.06 [0.89–1.24]	**0.79** [0.64–0.97]
Drinking		
No	*Reference*	*Reference*
Yes	**0.74** [0.61–0.89]	**0.51** [0.39–0.66]

Boldface values signify *p* < 0.05

Notably, a different pattern of urban-rural differences for functional limitations than for self-rated health was seen in Guangdong where the odds of poorer functional limitations for those living in rural areas was almost four times that of urban areas, but no statistically significant difference was observed for poor self-rated health. Covariates significantly associated with both adverse health outcomes were older age, poor income, no health insurance, and increasing number of chronic diseases. We undertook sensitivity analyses using different definitions for the two outcomes (see Appendix). Notably, compared to urban Shandong, rural Shandong remains with the largest disparity on (poor or very poor) self-rated health and functional limitations, followed by Jilin and Hubei. These findings were similar to the analyses reported in the main manuscript.

## Discussion

We found geographical variations in health not only between regions but also systematic differences by urban and rural areas, and with an interaction between these two geographical measures. Poorer health in most provinces is exacerbated for those in rural areas, highlighting the increased need for more health promotion and better health services outside of major cities. However, separate to the effect of rurality, health status among older persons was generally better in the Eastern region (Shanghai, Zhejiang, Guangdong and Shandong) compared to the Central and Western areas. Disparities between urban and rural areas varied across provinces. Notably, Shandong, which is in the Eastern region, and the two Central provinces (Jilin and Hubei) had the most pronounced adverse outcomes overall but also the greatest differences between urban and rural areas.

Other studies also reported that rural residents are subject to the double disadvantage of limited economic development and challenges of high risk factors (eg smoking), more chronic conditions (including chronic lower respiratory disease as well as stomach and liver cancers) and poorer access to health care [27–30]. These disparities warrant policy attention because older people in China live outside of megacities, with rural-urban migration of younger people contributing to acceleration of population ageing in rural areas. The health needs of older people may be left behind as development concentrates in cities in the more developed regions. Consequently, a large proportion of China’s older population, who will have high levels of comorbidity and need care and assistance with daily living, will be living in rural areas where health and social care is less readily accessible.

Our study demonstrated similar patterns of geographical variations as other national data in China. A cross-sectional study based on the Chinese National Health Services Survey 2008 using another health related quality of life measure (EQ-5D) reported worse outcomes in rural compared to urban residents across all regions, but disparities were more profound in Eastern areas [14]. The Chinese Health and Retirement Longitudinal Study and the Chinese Longitudinal Healthy Longevity Survey also both found substantial differences in health outcomes across provinces among older adults [31, 32]. Similar disparities in Shandong were also reported in another study noting that Shandong has the weakest social security and state influence, which could have an impact on the urban-rural inequalities in health [32].

Since 2011, China has made significant progress towards universal health coverage with basic medical insurance covering over 90% of urban residents and the new cooperative medical care enrolled over 95% of rural residents [33]. However, not having health insurance was associated with both adverse health outcomes in our study albeit vastly different levels of health insurance coverage. Basic medical insurance for urban residents and the new cooperative medical care has had a strong influence on health care utilisation of older Chinese [34–36]. Despite the high insurance coverage, urban-rural disparity in access to health services was associated with poor health outcomes among older adults and the role of health insurance is particularly important for households requiring inpatient hospital care [12, 19]. Besides differences in health insurance status, inequality in the distribution of health resources disfavouring rural areas has been noted in the literature [2, 10]. However, these health system factors were beyond the scope of our study and we are unable to control for these factors other than by the urban-rural classification.

Some findings on health covariates require further interpretation in relation to other international literature. For example, there were markedly different relationships between body mass index and self-rated health across East Asian countries – excess body mass index was negatively associated with poor self-rated health in China, however the reverse relationship was seen in Japan and South Korea [37]. Our findings of females reporting worse self-rated health were generally supported by other international studies [38], however, it is worth noting that there was a lower proportion of females in some rural areas in our study (Zhejiang, Guangdong, and Yunnan). There was also a smaller proportion of older females who smoke or consume alcohol in this dataset hence health promotion initiatives should take into account gender specific risk behaviours.

The strengths of our study include the WHO SAGE data, which are nationwide representative samples of older adults across key regions with comprehensive demographic and health information. Another feature of the data is the possibility of cross-country comparisons (in addition to China, other low and middle-income countries include Ghana, India, Mexico, Russian Federation and South Africa). Because our analyses are based on cross-sectional data, caution should be used on causal interpretation of the findings. Future comparative longitudinal evidence will be invaluable in monitoring overall health trends and inequalities among older populations. A number of determinants of health were not included in this study, for example, health inequalities are magnified by environmental conditions such as indoor and outdoor air pollution in China [39]. Differential health impacts on older adults across geographical areas could be a topic for future research employing spatial methods.

## Conclusion

Our study enhances the understanding of geographical variations in health outcomes among older Chinese revealing substantial disparities between urban and rural areas observed in both the well-developed areas (eg Shandong) and also the lower end of the economic spectrum (eg Hubei and Jilin). With rapid population ageing in China over the next few decades, it will be important to monitor the impacts of social and health policy at the national level but also by geographical areas. Designing appropriate social and health care policy should take into account geographical differences, for example, sources of support for rural persons may differ from those of urban areas and these can change substantially post retirement [40]. Gender-specific health interventions and targeted healthcare policies could minimise adverse health outcomes in later life.

## Appendix

**Table 3:** Selected socio-demographic indicators by provinces, 2015

Indicators	Shanghai	Zhejiang	Guangdong	Shandong	Jilin	Hubei	Shaanxi	Yunnan
Population (10,000 persons)	2,415	5,539	10,849	9,847	2,753	5,852	3,664	4,742
Male: female ratio (female=100)	108.4	107.4	113.5	104.5	102.0	104.1	107.5	105.0
Percent of population in urban areas	87.6	65.8	68.7	57.0	55.3	56.8	55.0	43.3
Average family size (persons/household)	2.46	2.69	3.23	2.88	2.92	3.05	3.08	3.49
Dependency ratio (% of 0-14 and 65+/15-64 years)	28.5	31.9	30.5	38.9	29.7	35.9	32.0	38.0
Percent illiterate population aged 15 and over	3.12	5.87	2.90	6.65	2.61	5.96	2.98	9.53
Per Capita Gross Regional Product (yuan)	103796	77644	67503	64168	51086	50654	34919	28806
Per Capita Household Consumption Expenditure	34784	24117	20976	14578	13764	14316	11729	11005
Number of community health service centres	306	467	1078	513	203	342	219	171
Number of inpatients (100 million person-times)	2.58	5.30	7.86	6.15	1.02	3.48	1.25	0.46
Number of inpatients (10,000 persons)	335	791	1442	1522	341	1108	381	749

Source: China Statistical Yearbook 2016, National Bureau of Statistics of China

**Table 4:** Geographic variations in self-rated health (comparing binary and multinomial outcomes), WHO SAGE China Wave 1

Explanatory variables	Logistic AOR [95% CI] Moderate/good/very good (Ref)	Multinomial AOR [95% CI] Good/very good (Reference)	
	Poor/very poor (21.4%) vs Reference	Poor/very poor (21.4%) vs Reference	Moderate (44.6%) vs Reference
Rural-urban interaction effects			
Rural Shanghai (*ref:* urban)	**1.82** [1.31-2.52]	**1.81** [1.25-2.62]	0.95 [0.75-1.20]
Rural Zhejiang (*ref:* urban)	**1.71** [1.18-2.46]	**1.66** [1.08-2.55]	0.98 [0.76-1.27]
Rural Guangdong (*ref:* urban)	0.78 [0.56-1.09]	**0.61** [0.41-0.92]	**0.44** [0.33-0.59]
Rural Shandong (*ref:* urban)	**6.32** [4.53-8.82]	**7.23** [4.87-10.8]	**1.20** [0.95-1.53]
Rural Jilin (*ref:* urban)	**2.71** [2.04-3.61]	**3.80** [2.65-5.45]	**1.49** [1.16-1.92]
Rural Hubei (*ref:* urban)	**2.36** [1.82-3.07]	**2.78** [1.97-3.93]	1.02 [0.76-1.37]
Rural Shaanxi (*ref:* urban)	**1.55** [1.17-2.06]	1.25 [0.66-1.39]	**0.62** [0.47-0.83]
Rural Yunnan (*ref:* urban)	0.97 [0.73-1.29]	0.96 [1.25-2.63]	0.90 [0.67-1.20]
Sociodemographic attributes			
Age groups in year			
50-59	*Reference*	*Reference*	*Reference*
60-69	1.07 [0.94-1.23]	1.10 [0.93-1.30]	**1.14** [1.01-1.28]
70+	**1.41** [1.22-1.63]	**1.76** [1.47-2.12]	**1.51** [1.32-1.73]
Sex			
Male	*Reference*	*Reference*	*Reference*
Female	**1.26** [1.10-1.45]	**1.23** [1.03-1.46]	**1.11** [0.98-1.25]
Years of education			
<6 years	*Reference*	*Reference*	*Reference*
≥6 years	1.11 [0.98-1.26]	1.05 [0.89-1.24]	0.99 [0.88-1.12]
Permanent income quintiles			
Quintile 1 (lowest)	**2.76** [2.18-3.49]	**4.48** [3.37-5.97]	**1.88** [1.55-2.29]
Quintile 2	**1.98** [1.58-2.49]	**2.51** [1.91-3.30]	**1.38** [1.15-1.65]
Quintile 3	**1.71** [1.37-2.14]	**2.11** [1.62-2.76]	**1.42** [1.17-1.69]
Quintile 4	**1.49** [1.20-1.86]	**1.68** [1.30-2.19]	**1.21** [1.04-1.42]
Quintile 5 (highest)	*Reference*	*Reference*	*Reference*
Health covariates			
Health insurance			
Mandatory and/or voluntary	*Reference*	*Reference*	*Reference*
No insurance	**1.22** [1.01-1.49]	1.15 [0.88-1.50]	0.93 [0.77-1.01]
Body mass index			
Underweight (<18.5)	**1.41** [1.12-1.82]	1.36 [0.98-1.88]	1.09 [0.84-1.42]
Normal (18.5 to 23.5)	*Reference*	*Reference*	*Reference*
Overweight (23.5 to 25.0)	0.92 [0.79-1.07]	**0.82** [0.68-0.99]	0.90 [0.79-1.03]
Obese (>25)	**0.68** [0.59-0.79]	**0.69** [0.58-0.82]	**0.89** [0.79-1.01]
Number of chronic diseases			
0	*Reference*	*Reference*	*Reference*
1	**2.47** [2.16-2.83]	**3.78** [3.20-4.46]	**2.05** [1.83-2.31]
2+	**4.80** [4.13-5.58]	**12.7** [10.3-15.7]	**3.46** [2.96-4.06]
Smoking			
No	*Reference*	*Reference*	*Reference*
Yes	1.06 [0.89-1.24]	**0.92** [0.75-1.13]	0.88 [0.77-1.01]
Drinking			
No	*Reference*	*Reference*	*Reference*
Yes	**0.74** [0.61-0.89]	**0.62** [0.50-0.77]	**0.82** [0.70-0.94]

Boldface values signify *p* < 0.05

**Table 5:** Geographic variations in functional limitations (comparing two cut-offs) WHO SAGE China Wave 1

Explanatory variables	Logistic AOR [95% CI] based on WHO DAS scores	
	Scores≥25 (12.5%) Moderate and over	Scores≥12.5 (25.3%) Minor
Rural vs Urban (reference)		
Interaction effects		
Shanghai (ref: urban)	1.31 [0.85-2.01]	**1.99** [1.43-2.77]
Zhejiang (ref: urban)	0.69 [0.45-1.09]	**0.66** [0.47-0.90]
Guangdong (ref: urban)	**3.69** [2.43-5.59]	**3.57** [2.58-4.94]
Shandong (ref: urban)	**5.14** [3.55-7.44]	**7.75** [5.78-10.4]
Jilin (ref: urban)	**4.72** [3.43-6.49]	**5.81** [4.51-7.49]
Hubei (ref: urban)	**4.11** [2.80-6.04]	**5.14** [3.84-6.89]
Shaanxi (ref: urban)	1.33 [0.93-1.90]	0.76 [0.58-1.00]
Yunnan(ref: urban)	**1.88** [1.29-2.74]	**1.78** [1.29-2.47]
Sociodemographic attributes		
Age groups in year		
50-59	*Reference*	*Reference*
60-69	**1.61** [1.33-1.96]	**1.55** [1.35-1.78]
70+	**5.25** [4.34-6.35]	**4.97** [4.30-5.73]
Sex		
Male	*Reference*	*Reference*
Female	1.14 [0.97-1.34]	**1.37** [1.20-1.57]
Years of education		
<6 years	*Reference*	*Reference*
≥6 years	**0.80** [0.68-0.95]	**0.88** [0.77-0.99]
Permanent income quintiles		
Quintile 1 (lowest)	**2.88** [2.09-3.97]	**2.69** [2.09-3.97]
Quintile 2	**2.06** [1.49-2.85]	**1.73** [1.37-2.18]
Quintile 3	**1.83** [1.34-2.49]	**1.48** [1.18-1.85]
Quintile 4	**1.62** [1.20-2.18]	**1.44** [1.16-1.79]
Quintile 5 (highest)	*Reference*	*Reference*
Health covariates		
Health insurance		
Mandatory and/or voluntary	*Reference*	*Reference*
No insurance	**1.36** [1.06-1.76]	1.17 [0.96-1.42]
Body mass index		
Underweight (<18.5)	1.16 [0.84-1.61]	**1.41** [1.09-1.83]
Normal (18.5 to 23.5)	*Reference*	*Reference*
Overweight (23.5 to 25.0)	0.97 [0.80-1.19]	0.89 [0.77-1.04]
Obese (>25)	**1.21** [1.02-1.43]	1.05 [0.92-1.21]
Number of chronic diseases		
0	*Reference*	*Reference*
1	**1.74** [1.46-2.08]	**1.74** [1.52-1.99]
2+	**3.34** [2.81-3.96]	**3.86** [3.33-4.47]
Smoking		
No	*Reference*	*Reference*
Yes	**0.79** [0.64-0.97]	**0.87** [0.74-1.02]
Drinking		
No	*Reference*	*Reference*
Yes	**0.51** [0.39-0.66]	**0.68** [0.57-0.81]

Boldface values signify *p* < 0.05

## Abbreviations

AOR: Adjusted odds ratio
CI: Confidence interval
SAGE: Study on global ageing and adult health
WHO: Disability Assessment Schedule 2.0 (WHODAS 2.0)
